# Monthly Summary

**Published:** 1878-12

**Authors:** 


### MONTHLY SUMMARY.

---

*Turpentine Vapor in Accidents from Chloroform Vapor.*—Dr. Wachsmuth, of Berlin, has suggested the use of turpentine vapor as a preventive of those accidents which frequently occur in the administration of chloroform to produce anæsthesia. He says that it is well known to every physician that death often takes place suddenly from the vapor of chloroform, in spite of the greatest care and the use of every precaution before and during its administration. The operator, even when assisted by three or four of his colleagues, may see his patients die before him. There is, he states, a very easy and simple remedy for preventing the occurrence of such a serious accident. It consists in the addition of one part of rectified oil of turpentine to five parts of chloroform. The oil of turpentine in vapor appears to exert a stimulating or life-giving effect on the lungs, and protects these organs from passing into that paralyzed state which seems to be produced by chloroform-narcosis. Dr. Wachsmuth, while lying on a sick bed, accidentally breathed the vapor of turpentine, and he experienced from this a strongly refreshing feeling. This fact induced him to try the plan of adding oil of turpentine to chloroform when the latter was used for anæsthetic purposes. The beneficial results surpassed his expectation.—*London Med. Record.*

---

*A Case of Third Dentition.*—This case is reported in the *British Med. and Surg. Jour.* The subject had his four upper incisors extracted on account of neuralgic pains, when he was 47 years of age. Ten years afterwards the two upper teeth on the left side reappeared; they were smooth, thin, and transparent, and soon become loose. Two years later they were pushed out with the finger without difficulty.—*Med. Record.*

